# Supplementary material for: Heart Failure Association-International Cardio-Oncology Society Risk Score Validation in HER2-Positive Breast Cancer
Source: J Clin Med. 2023 Feb 6;12(4):1278. doi: 10.3390/jcm12041278 (PMC9963986; doi:10.3390/jcm12041278)

1 - Unadjusted Patient Characteristics

**Characteristics**	**Overall (N=507)**	**Age≤ 65 (N=374)**	**Age≥ 65 (N=133)**
**Continuous variables**			
Age (Years)			
Median	55	51	75
IQR	48-66	45-57.5	70-79
Mean	57	50	75
Standard Deviation	13.7	8.8	7
Range	23-95	23-64	65-95

**Characteristics**	**Overall (N)**	**%**	**Age≤ 65 (N)**	**%**	**Age≥ 65 (N)**	**%**
**Categorical variables**						
Sex						
Female	507	100	374	100	133	100
Male	0	0	0	0	0	0
Comorbidities						
Diabetes	23	4.6	14	3.7	9	6.9
Hypertension	106	20.9	52	13.9	54	40.6
Kidney disease	31	6.1	11	2.9	20	15.2
Coronary Artery Disease	0	0	0	0	0	0
Cardiomyopathy	1	0.2	1	0.3	0	0
Peripheral vascular disease	0	0	0	0	0	0
CHF	17	3.4	5	1.3	12	9
Smoking	106	20.9	81	21.6	25	18.8
Status						
Dead	236	46.5	159	42.5	77	57.9
Alive	270	53.3	214	57.2	56	42.1
Grade						
0	26	5.1	18	4.8	8	6
1	5	1	2	0.5	3	2.3
2	183	36.1	138	36.9	45	33.8

3	293	57.8	216	57.8	77	57.9
Previous trastuzumab use	375	74	297	79.4	78	58.6
Previous anthracycline use	87	17.2	79	21.1	8	6
ER Status						
Positive	302	59.6	222	59.4	80	60.2
Negative	202	39.8	150	40.1	52	39
PgR Status						
Positive	263	51.9	197	52.7	66	49.6
Negative	240	47.3	175	46.8	65	48.9
Histology						
Ductal	421	83	313	83.7	108	81.2
Lobular	24	4.7	15	4	9	6.8
Mixed	4	0.4	2	0.5	2	1.5
Other	58	11.5	44	11.8	14	10.5
Concurrent Therapy						
Diuretics	0	0	0	0	0	0
Betablockers	52	10.3	18	4.8	34	25.6
Statins	85	16.8	37	9.9	48	36.1
Anticoagulant	22	4.3	10	2.7	12	9
Calcium antagonists	0	0	0	0	0	0
ACE inhibitors	63	12.4	31	8.3	32	24.1
Angiotensin receptor blockers	63	12.4	31	8.3	32	24.1

2 - ROC Graph Related to Treatment

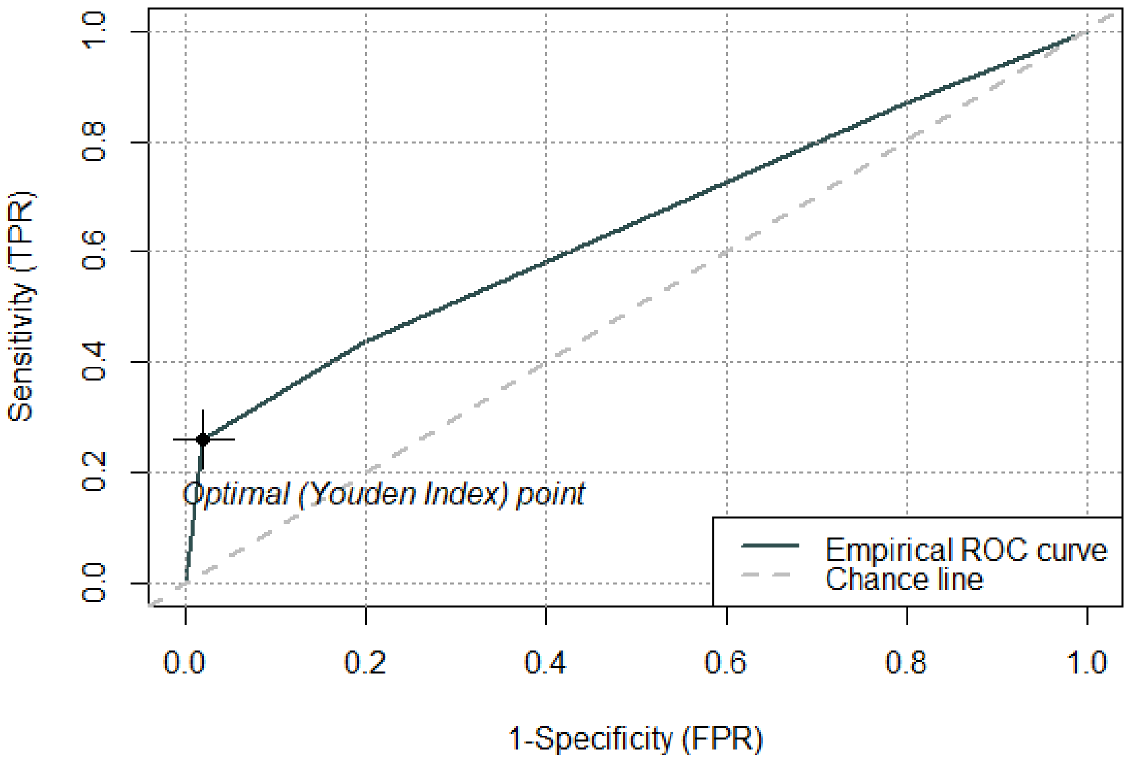

3 - ROC graph relating to death

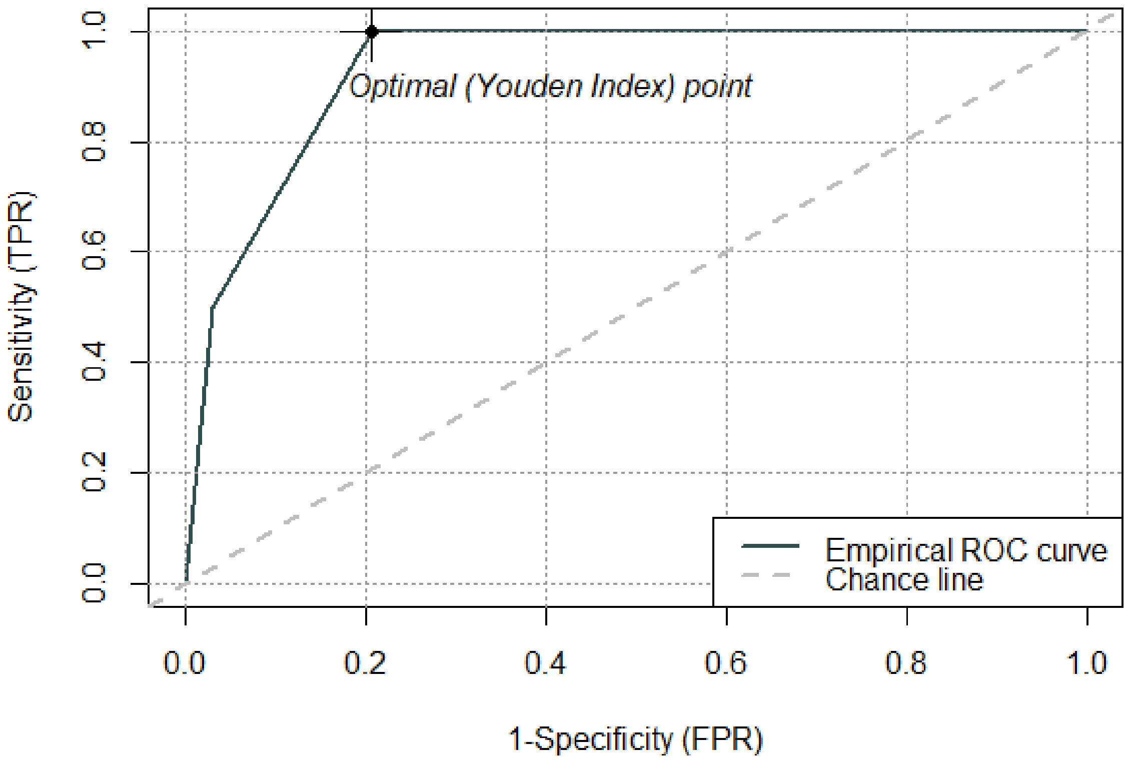

4 - ROC Graph relating to drop in LVEF <50%

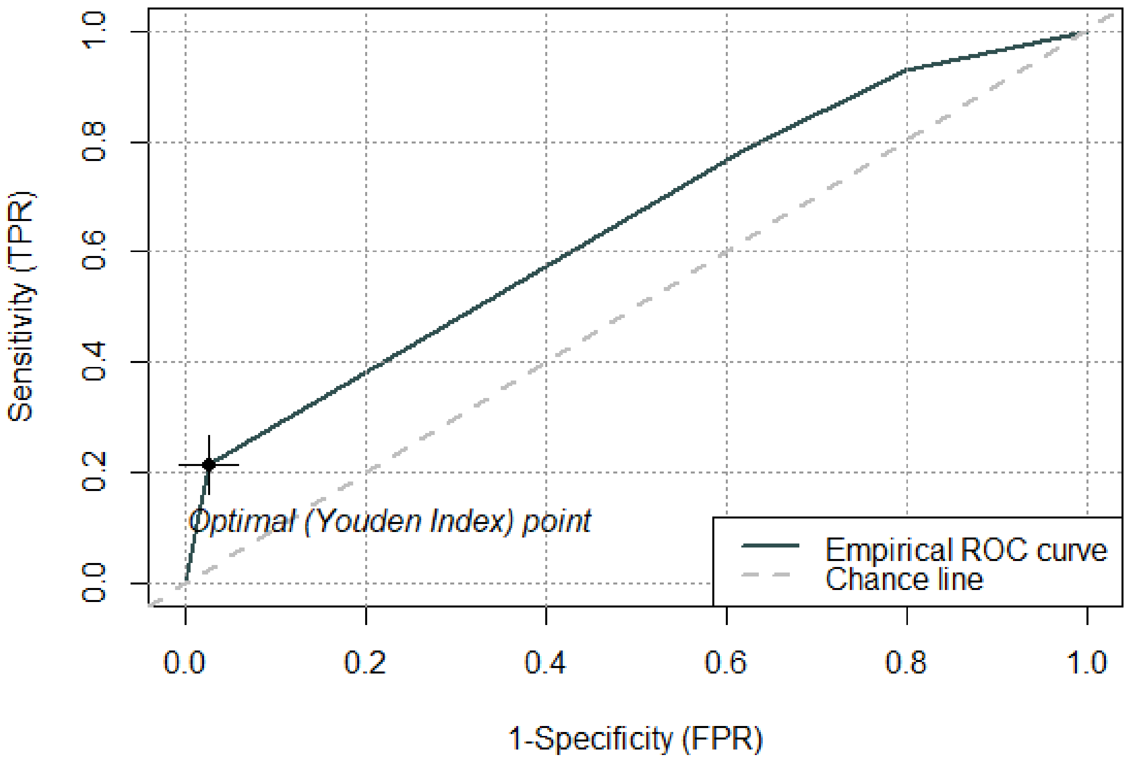

Supplement: Supplementary file 1 [file jcm-12-01278-s001.zip › jcm-2137142-supplementary.pdf]
